# Validity of the Parental Burnout Inventory Among Dutch Employees

**DOI:** 10.3389/fpsyg.2018.00697

**Published:** 2018-05-23

**Authors:** Hedwig J. A. Van Bakel, Marloes L. Van Engen, Pascale Peters

**Affiliations:** ^1^Department of Tranzo, Tilburg School of Social and Behavioral Sciences, Tilburg University, Tilburg, Netherlands; ^2^Department of Human Resource Studies, Tilburg School of Social and Behavioral Sciences, Tilburg University, Tilburg, Netherlands; ^3^Institute for Management Research (IMR), Radboud University Nijmegen, Nijmegen, Netherlands

**Keywords:** parental burnout, Dutch working parents, Parental Burnout Inventory, employees, parental stress, validation study

## Abstract

The purpose of this study was to validate the Parental Burnout Inventory (PBI) in a Dutch sample of working parents. The Dutch version of the PBI and questionnaires about work were administered to 627 working parents, with at least one child living at home. We investigated whether the tri-dimensional structure of the PBI held in a sample of male and female employed parents. Furthermore, we examined the relationships between PBI and the constructs work-related burnout, depressive mood, parenting stress and work-family conflict, which we assessed with widely used and validated instruments, i.e., emotional exhaustion [a subscale of the Dutch version of Maslach’s Burnout Inventory], a Dutch Parental Stress Questionnaire and Work-Family Conflict. The results support the validity of a tri-dimensional parental burnout syndrome, including exhaustion, distancing and inefficacy. Low to moderate correlations between parents’ burnout symptoms and professional exhaustion, parenting stress, depressive complaints and work-family conflict experiences were found, suggesting that the concept of PBI differs significantly from the concepts of job burnout, depression and stress, respectively. The current study confirms that some parents are extremely exhausted by their parental role. However, the number of Dutch employees reporting extreme parental burnout is rather low.

## Introduction

Nowadays, work-related stress is among the main causes of occupational diseases (Van Zwieten et al., 2014). In the Netherlands, for example, 13% of employees are dealing with burnout complaints (Smulders et al., 2013). Generally, work-related burn-out particularly accounts for individuals who have to fulfill both professional and parental responsibilities (Kossek and Lautsch, 2012). These individuals are seriously facing the challenge of participating successfully both as employee and family member, since involvement in any role is associated with additional demands on working hours, time allocated to care for children, and time for household tasks (Prottas and Hyland, 2011). Usually, not all demands can be fully met and the accumulation of roles may call upon an individual’s scarce resources, such as time, energy and commitment, and may enhance the experience of stress (Hobfoll, 2001; Eby et al., 2005; Bakker and Demerouti, 2007). As a result of this accumulation of enduring, excessive role demands and associated stress, more and more people suffer from burnout (Weber and Jaekel-Reinhard, 2000; Maslach et al., 2001; Brewer and Shapard, 2004).

Burnout has primarily been described as a psychological syndrome originating from the work domain (Maslach, 1993). However, recently researchers have published about the existence of a related phenomenon which can be linked to increased levels of stress: parental burnout (Norberg, 2007; Lindström et al., 2011; Roskam et al., 2017). The essence of this recently described construct is that, in the same way as that people can experience burnout from their job, they can experience burnout from their parental role. In their validation study with French speaking parents, Roskam et al. (2017) confirmed that parental burnout is a unique, specific syndrome that differs from the well-known concept work-related burnout. This has led to the definition of parental burnout as being a psychological syndrome that manifests itself in the parental context and which is characterized by the following three key dimensions related to the role of parenting: (1) exhaustion in taking care of children; (2) emotional distancing from children; and (3) low personal accomplishment with regard to parenting.

Similar to work-related burnout, the first dimension of parental burnout, exhaustion, is considered to be the core dimension of this latter concept. It entails feelings of being overloaded and depleted of one’s physical and emotional resources caused by parenting. Parents feel too stressed to care of their children and feel that being a parent requires too much involvement. The second dimension of parental burn-out, emotional distancing, refers to a situation in which a parent disengages emotionally (rather than physically) from their children in order to distance oneself from the source of exhaustion. This implies that a parent physically still provides care for his or her child(ren) [e.g., bringing the child(ren) to bed or feeding them], but becomes less emotionally involved and thereby less sensitive and responsive to the child(ren)’s signals and needs. The third dimension of parental burnout, personal accomplishment, refers to a parent’s feeling of incompetence as a parent and to a lack of achievement in the parental role (Roskam et al., 2017). They feel that they are unable to accomplish worthwhile things as a parent.

In recent years, a small number of studies have looked into factors affecting parental burnout. In parallel with the Job-Demands Resource Theory (Bakker and Demerouti, 2014) that makes a distinction between demands and resources characterizing the work domain (i.e., organization, job and personal factors), several factors characterizing the parenting domain can be distinguished that can affect parental burnout. For example, particularities of children that need care, such as having a chronic or serious disease, may result in higher levels of exhaustion in their parents (Norberg, 2007; Lindström et al., 2010). However, Mikolajczak et al. (2017) found that care demands, such as particularities of children (e.g., displaying behavior problems or having a disability/chronic illness), explained less variance in parental burnout than parents’ stable traits and family functioning, which can be regarded as (a lack of) parents’ personal resources. For example, in a non-randomly selected sample, Le Vigouroux et al. (2017) showed that particularly stable parental personality characteristics, such as high neuroticism and low levels of conscientiousness and agreeableness, significantly affected parental burnout.

Moreover, parents’ experienced work-family conflicts can be viewed a factor in parental burnout. Previous research already showed that employees who are confronted with work overload and emotional demands at work have more problems combining work with their family life (Bakker and Geurts, 2004; Butler et al., 2005). These conflicts between the work and family domain subsequently have been found related to psychological well-being and job burnout (Geurts et al., 1999; Rupert et al., 2009). In a similar vein, it can be argued that work-family conflict at the interface of both the work and the non-work domains can be related to parental burnout. Hence, when demands in the work or family domains come into conflict, resources in the family domain may be lost or threatened and may increase the degree of parental burnout.

The aim of the current study is to examine the validity of the Dutch translation of the Parental Burnout Inventory (PBI) by replicating the work of Roskam et al. (2017) among male and female employees with children living at home in the Netherlands. To this end, we first assessed whether the tri-dimensional structure of the PBI (i.e., exhaustion, depersonalization and emotional distancing) could also be found in a sample of Dutch employees. Next, we tested the relationship between parental burnout and work-related burnout (i.e., emotional exhaustion), depressive mood, parenting problems and work-family conflict, respectively. We also examined preliminary cut-offs and the number of parents reporting parental burnout in the Dutch context.

## Methodology

### Sample and Procedure

The data used in this validation study was collected using self-administered paper-and-pencil questionnaires. To generate the study sample, a convenience sampling technique was used (Etikan et al., 2016). Respondents were approached based on their proximity and accessibility to the data collectors, i.e., 3rd year Bachelor students participating in a research course and master students of Tilburg University. Parents of infants, preschool age children, primary school aged children, secondary education aged children and older children were sampled. Participants had to meet the following two inclusion criteria: (1) to work as an employee for at least 12 h a week and; (2) to be a parent and have at least one child living at home.

### Data Analyses

In the current paper, we examined the internal structure of the PBI and its relations to other employee outcomes. With regard to the internal structure of the PBI, principal components analyses (PCA), parallel analyses and confirmatory factor analyses (CFA) were performed to test if the three-factor structure as described by Roskam et al. (2017) could be replicated in our sample of Dutch employees. Reliability was estimated with Cronbach’s alpha coefficients. The measurement models included the three latent variables representing the concepts of emotional exhaustion (eight items), emotional distancing (eight items) and personal accomplishment (six items). Analyses were conducted based on the covariance matrix and using maximum likelihood estimation. Goodness-of-fit indices, χ^2^, the comparative fit index (CFI) and the root-mean-square error of approximation (RMSEA), were used to determine the acceptability of the model.

To investigate the relationships between the parental burnout construct and the employee outcome variables salient to our study (to estimate the uniqueness of the PBO construct), correlation coefficients were examined with work-related burnout, depressive mood, parenting stress and work-family conflict. In accordance with Roskam et al. (2017), the prevalence of parental burnout in Dutch parents was estimated with three concurrent methods.

### Participants

In total 677 questionnaires were returned. To be included in the current study, individuals had to meet the following two inclusion criteria: (1) to work as an employee for at least 12 h a week and; (2) to be a parent and have at least one child living at home. Based on these stratification criteria, 50 respondents had to be excluded from the final sample used for the present study [based on the number of working hours a week (10 missing; 2 working less than 12 h a week); number of children living at home (1 missing; 25 no children living at home); missing demographic information (12 missing)]. The final sample consisted of 627 respondents (all having a Dutch nationality), including 252 male and 375 female respondents. Their average age was 44.87, ranging from 23 years old to 66 years old (*SD* = 8.48). Furthermore, the number of children living at home ranged from 1 to 5 (*M* = 1.92) and the average number of weekly working hours was 34.72 (*SD* = 10.70). In addition, 91.7% of the respondents was married or cohabiting and 8.3% of the respondents was single, divorced or widowed.

### Measures

#### Parental Burnout

To measure parental burnout, the final 22-item version of the PBI as presented by Roskam et al. (2017) was used. First, three experts (and three master students) were independently involved with the translation process (Maneesriwongul and Dixon, 2004). Two experts translated the scale from English/French into Dutch. Results were checked by a third expert. Moreover, three master students compared the translation with the original formulation of the French (and English) PBI (Roskam et al., 2017). Consensus was found on all items. The Dutch translation was checked and approved by the developers. Additional permission for copyright was acquired from Mind Garden Inc. for the use of the PBI.

The original 22-item version of the PBI consisted of three theoretical dimensions ‘emotional exhaustion’ (EE: eight items) “*I feel my parental role is breaking me down*,” ‘emotional distancing’ (ED: eight items) “*I do not really listen to what my children tell me*,” and ‘personal accomplishment’ (PA: six items) “*I look after my children’s problems very effectively.*” Respondents had to indicate how often each of the 22 statements were applicable to them. The seven-point Likert scale response categories ranged from 0 (never) to 6 (every day), with six items reverse-scored, which was also used in Roskam et al. (2017). Higher scores indicate higher levels of parental burnout. The construct validity and reliability of the PBI is discussed under the heading ‘results.’

#### Work-Related Burnout

Work-related burnout was assessed with the subscale ‘emotional exhaustion’ of the Dutch version of the Maslach Burnout Inventory (MBI) (Schaufeli and van Dierendonck, 2000). The MBI is a widely used 22-item questionnaire originally encompassing three factors (i.e., emotional exhaustion, depersonalization, and personal accomplishment). An example item of the emotional exhaustion scale (eight items) is for example “*I feel emotionally drained from my work*.” Participants were asked to rate each item on a five-point Likert scale ranging from 1 (never) to 5 (very often). The global total score for work-related burnout (i.e., emotional exhaustion) was computed as the mean score of all eight items. Higher scores indicate greater work-related burnout. The Cronbach’s alpha for this subscale reported in the MBI manual was 0.90. The Cronbach’s alpha in the current study is 0.89.

#### Parental Stress and Depressive Mood

Parental stress and parent depressive mood were assessed with the original 10-item version of the Parental Stress Questionnaire (PSQ-s) (Vermulst et al., 2015). Items were rated on a four-point Likert scale from ‘not true’ (1) to ‘very true’ (4). The abbreviated 10-item version included items from the original 34-item: ‘parent-child relationship problems’ (3 items); ‘parenting problems’ (4 items); and ‘depressive mood’ (3 items). To test the underlying structure of the abbreviated parental stress scale, an exploratory factor analysis was conducted. Considering Kaisers’ criterion, two components had Eigenvalues above 1 and also the Cattell’s scree plot identified two components. The first factor consisted of ‘parent–child relationship and parenting problems’ (7 items) and the second factor consisted of 3 items representing ‘depressive mood.’ Therefore, two subscales (‘parent–child parenting problems’ and ‘depressive mood’) were created as well as a total scale (‘parenting stress’) by using the sum scores on the corresponding items. Cronbach’s alphas found in the current sample were 0.86, 0.77, and 0.89 for the subscales and the total scale, respectively.

#### Work-Family Conflict

Work-family conflict was evaluated with 12 items developed by Carlson et al. (2000). The scale assesses to what extent work negatively influenced the home situation or family activities and *vice versa*. Example items are “*The time I must devote to my job keeps me from participating in household responsibilities and activities*” and “*I am often so emotionally drained when I get home from work that it prevents me from contributing to my family*” and participants were asked to rate each item on a 5-point Likert scale ranging from 1 (strongly disagree) to 5 (strongly agree). Principal Component Analysis showed four underlying factors of work-family conflict, two factors consisting of work-to-family conflict (time-based and strain-based) and two factors about family-to-work conflict (time-based and strain-based), which also have been found and explained in the work-family conflict theory (Carlson et al., 2000). Based on the PCA and theory, two mean scores were computed (Geurts et al., 2005), work-to-family conflict (6 items) and family-to-work conflict (6 items). Cronbach’s alphas of the two scales were 0.80 and 0.81, respectively.

#### Demographics

In addition, participants were asked about their gender, age, nationality, marital status (married, cohabiting, single, divorced, or widowed), level of education, number of children living at home and weekly working hours. Although our sample characteristics were similar to Roskam et al.’s (2017) samples concerning the parents’ age, educational level and marital status, our sample consisted of 60% women, whereas Roskam’s samples consisted of far higher percentages of women (i.e., 83 and 87%).

## Results

### Factor Structure and Reliability

Means (*M*) and standard deviations (*SD*) of the demographic study variables are presented in **Table 1**.

**Table 1:** Characteristics of the study sample (*N* = 627).

		*N* (%)	*M*	*SD*	Range
(1) Gender				
Men		252 (40.2%)			
Women		375 (59.8%)			
(2) Parent’s age		576	44.85	8.48	23–66
(3) Marital status				
Married/Partnered		575 (91.7%)			
Single parent		52 (8.3%)			
(4) Parental educational level
12 years		82 (13.2%)			
12–15 years		230 (37%)			
>15 years		310 (49.4%)			
(5) Number of children living at home		627	1.92	0.77	1–5
(6) Age of the youngest child living at home		627			0–31

To test its underlying structure, the 22 items of the PBI were first subjected to principal components analysis (PCA) using SPSS version 22. Prior to performing PCA, the suitability of the data for factor analysis was assessed. Inspection of the correlation matrix revealed the presence of many coefficients of 0.3 and above. The Kaiser-Meyer-Olkin value was 0.91, exceeding the recommended value of 0.6 and Bartlett’s Test of Sphericity reached statistical significance, supporting the factorability of the correlation matrix.

Principal components analyses revealed the presence of four components with eigenvalues exceeding 1, explaining 36.8% (eigenvalue 8.10), 13.7% (eigenvalue 3.01), 9% (eigenvalue 1.97), and 4.6% (eigenvalue 1.01) of the variance, respectively. An inspection of the screeplot, however, revealed a clear break after the third component. Using Cattell’s scree test, it was decided to retain three components for further investigation. The three factor structure was further supported by the results of Parallel Analysis, which showed only three components with eigenvalues exceeding the corresponding criterion values for a randomly generated data matrix of the same size (22 variables × 627 respondents; eigenvalues 1.35, 1.29, 1.25, and 1.21, respectively). The subsequent three-component solution explained a total of 59.44% of the variance, with Component 1 contributing 36.8%, Component 2 contributing 13.7% and Component 3 contributing 9%. For the interpretation of these three components, Oblimin rotation was performed. The rotated solution revealed the presence of simple structure, with all three components showing a number of strong loadings and all variables loading substantially on only one component. The interpretation of the three components was consistent with previous research on the PBI (Roskam et al., 2017). The EE items loaded strongly on Component 1, the ED items loaded strongly on Component 2, and the PA items strongly loaded on Component 3. Moreover, weak negative correlations between the three factors were found (*r* = -0.25, -0.47, and 0.41, respectively). The results of these analyses supported the use of the EE items, ED items and PA items to be used as separate scales (cf. Roskam et al., 2017).

In order to conduct Confirmatory Factor Analysis (CFA), using AMOS 22, all items were first log transformed. Skewness and kurtosis indicated that the items of the PBI, as expected, displayed deviations from normality. CFA was performed twice, once including original item scores and once including transformed items. Since estimates and model fit indices were similar only results obtained from the analyses computed on scores of original variables were presented. The Chi-square [χ^2^_(206)_ = 1029.1, *p* < 0.001] suggests a significant portion of the variance being not explained by the model. However, other fit indices demonstrated sufficient fit to the data and acceptability of the model (CFI = 0.89, RMSEA = 0.08) with all estimated factor loadings being significant *p* < 0.001. The standardized factor loadings are listed in **Table 2**. Correlations between the three factors were 0.58 (ED-EE), 0.52 (ED-PA), and 0.36 (PA-EE), respectively. These results further provide support for the validity and internal structure of the Dutch translation of the PBI.

**Table 2:** Standardized regression weights from confirmatory factor analysis for the 22-item version of the Dutch translation of the PBI.

	Emotional distancing	Emotional exhaustion	Decreased personal accomplishment
ED1	0.590		
ED2	0.670		
ED3	0.695		
ED4	0.779		
ED5	0.805		
ED6	0.690		
ED7	0.771		
ED8	0.645		
EE1		0.688	
EE2		0.636	
EE3		0.655	
EE4		0.764	
EE5		0.596	
EE6		0.757	
EE7		0.699	
EE8		0.605	
PA1			0.682
PA2			0.824
PA3			0.863
PA4			0.843
PA5			0.818
PA6			0.796

Finally, reliability analyses were performed to test the consistency of all items in the EE, ED, and PA subscales and in the overall scale, with Cronbach’s alphas of 0.88, 0.87, 0.92, and 0.91, respectively. This also suggests good internal consistency reliability for the (sub)scales in our sample.

### Relationships With Other Variables

To examine the relationships between the PBI and salient variables (work-related burnout, depressive mood, parenting problems and work-family conflict), scores were obtained by summing the item scores of the three subscales (reverse-scored for personal accomplishment). Descriptive statistics and mean scores and correlations between the PBI (sub)scales and work-related burnout, depressive mood, parenting problems and work-family conflict are presented in **Table 3**. Due to deviations in normality, non-parametric correlations were computed. Low to moderate coefficients suggest significant relationships between parental burnout, work-related burnout, depressive mood and work-family conflict. No complete overlap was shown between the parental burnout concepts and the other study variables. The small correlation coefficient with work-family conflict suggests that parental burnout is not just a work-family conflict issue. An additional principal component analysis, with all items of the PBI (22 items), work-related burnout (8 items), depressive mood (3 items), parenting problems (7 items) and work-family conflict (12 items) entered into a common factor analysis followed by varimax rotation, revealed ten factors with eigenvalues of above 1.00; 1.00 (PBI-EE, 3 items), 2.55 (PBI-EE, 5 items), 5.72 (PBI-ED), 3.12 (PBI-PA), 12.08 (work related burnout), 1.06 (depressive mood), 2.67 (parenting stress), 1.44 (Work-family conflict time), 1.34 (Family-work conflict time), 2.01 (Family-work conflict strain). Work-family conflict strain items loaded on the first factor work-related burnout.

**Table 3:** Correlations among study variables.

	*M*	*SD*	Range	1	2	3	4	5	6	7	8	9	10
(1) PBI EE	4.27	5.06	0–30	–									
(2) PBI PA	5.66	6.15	0–36	0.30^∗∗^	–								
(3) PBI ED	5.58	6.54	0–41	0.52^∗∗^	0.47^∗∗^	–							
(4) PBI TOTAL	15.41	13.93	0–93	0.73^∗∗^	0.80^∗∗^	0.86^∗∗^	–						
(5) Work-related burnout	17.84	4.90	8–39	0.39^∗∗^	0.11^∗^	0.27^∗∗^	0.29^∗∗^	–					
(6) Depressive mood	4.91	1.64	3–11	0.37^∗∗^	0.38^∗∗^	0.35^∗∗^	0.44^∗∗^	0.35^∗∗^	–				
(7) Parenting problems	12.20	3.27	7–24	0.38^∗∗^	0.50^∗∗^	0.40^∗∗^	0.54^∗∗^	0.16^∗∗^	0.62^∗∗^	–			
(8) Parental stress	17.12	4.47	10–33	0.41^∗∗^	0.51^∗∗^	0.42^∗∗^	0.56^∗∗^	0.25^∗∗^	0.82^∗∗^	0.96^∗∗^	–		
(9) Work-Family conflict	16.64	2.30	11–24	0.23^∗∗^	0.08	0.18^∗∗^	0.19^∗∗^	0.56^∗∗^	0.26^∗∗^	0.11^∗^	0.17^∗∗^	–	
(10) Family-Work conflict	11.68	2.97	6–24	0.26^∗∗^	0.12^∗^	0.17^∗∗^	0.24^∗∗^	0.27^∗∗^	0.28^∗∗^	0.19^∗∗^	0.24^∗∗^	0.36^∗∗^	–

*N = 548–627. ^∗^p < 0.01, ^∗∗^p < 0.001*.

### Differences Between Fathers and Mothers

As can be seen in **Table 4**, the fathers reported significantly more parental burnout symptoms in comparison with the mothers in our sample. Fathers particularly reported more emotional distancing and lower levels of personal accomplishment than mothers. In contrast, women reported significantly more emotional exhaustion in their professional jobs (work-related burnout) and experienced more family-to-work-conflict than men. Levels of depressive symptoms and parental stress did not differ between men and women.

**Table 4:** Means and standard deviations of parental burnout, work-related burnout, parenting stress and work-family conflict for fathers and mothers.

	Fathers		Mothers		
	*M*	*SD*	*M*	*SD*	*t*
(1) PBI EE	4.17	5.24	4.35	4.94	-0.43
(2) PBI PA (decreased)	6.94	6.80	4.80	5.53	4.11**
(3) PBI ED	6.75	6.99	4.79	6.11	3.59**
(4) PBI TOTAL	17.64	14.38	13.89	13.43	3.21*
(5) Work-related burnout	17.09	4.80	18.34	4.9	-3.13*
(6) Depressive mood	5.02	1.60	4.83	1.67	1.38
(7) Parenting problems	12.28	3.16	12.15	3.34	0.46
(8) Parental stress total	17.29	4.34	17.01	4.57	0.78
(9) Work-Family conflict	16.44	2.22	16.78	2.34	-1.78
(10) Family-Work conflict	11.08	2.92	12.09	2.94	-4.21**

*N = 548–627. ^∗^p < 0.01, ^∗∗^p < 0.001.*

### Prevalence of Parental Burnout

The prevalence of parental burnout as assessed with the Dutch translation of the PBI was estimated according to the constructs described by Roskam et al. (2017). First, according to the cutoff points provided for work-related burnout, (Maslach et al., 2010) and Roskam et al. (2017), 87.5% of the parents (91.2% of the mothers and 82% of the fathers) belonged to the ‘low parental burnout’ category (score < 30); 10.4% of the parents (6.7% of the mothers and 15.9% of the fathers) to the ‘average parental burnout’ category (score 31–54); and 2.1% of the parents (2.1% of the mothers and 2% of the fathers) to the ‘high parental burnout’ category (score > 55). According to the theoretical approach, based on the response scale (ranging from 0 to 6 for each statement and a total scale score between 0 and 132) considering scores over 88 (experiencing burnout symptoms at least once a week), 0.2% of the parents (0.3% of the mothers and 0.0% of the fathers) can be considered to experience a parental burn out. Using a theoretical cutoff of 67 [experiencing most symptoms of parental burnout at least a few times a month (3)] revealed a prevalence score of 1.2% (1.7% of the mothers and 0.4% fathers). Considering burnout as a statistical cutoff corresponding to 1.5 the standard deviation above the mean of the current sample, 7.7% of the parents (5.7% of the mothers and 10.8% of the fathers) can be considered burned out by parenting. Subsequent analyses showed that the number of children significantly differed between parents who scored above the statistical cutoff (*M* = 2.15, *SD* = 0.84) and parents who scored below the statistical cutoff of 1.5 standard deviation above the mean (*M* = 1.89, *SD* = 0.76), *t* = 2.22, *p* < 0.05. Marital status, age of the children and number of working hours per week were not significantly related to the cutoff scores. Because of controversy in terms of possible cut off points, we need to be cautious in reporting and using the preliminary prevalence rates.

## Discussion

The aim of the present study was to examine the construct validity of our Dutch translation of the PBI (Roskam et al., 2017) in men and women who combine a professional job with parenting tasks. In this concluding section, the results of our empirical study will be summarized and discussed. Moreover, the implications of our findings for future research and policy will be outlined.

### Reflection Upon the Outcomes

First, this study supported the tri-dimensional structure of parental burnout (Roskam et al., 2017) in the Dutch context. In parenting employees in the Netherlands, women as well men, the separate constructs of emotional exhaustion, emotional distancing, and decreased personal accomplishment were found, which provides support for the generalizability of the results by Roskam et al. (2017) for the French speaking context to the Dutch context. Moreover, the low to moderate correlations between parental burnout and work-related burnout, parental stress and depressive mood suggest that significant relationships exist between those constructs, but that the aforementioned concepts are not interchangeable. More specifically, this underlines that the concept of parental burnout can be viewed a different concept than the well-known and widely studied concept of work-related burnout (Maslach and Leiter, 2016). This is also supported by the finding that men reported significantly higher parental burnout scores than women, whereas this latter group reports significantly higher work-related burnout. The independence between those constructs was also supported by additional factor analyses, including work-related burnout items, PBI items, parental stress items and work-family conflict items. These analyses revealed separate factors.

Second, in line with Roskam et al. (2017), we found the same three subdimensions in the overall concept of parental burn-out. This suggests that parental burnout, like work-related burnout, can be described in terms of exhaustion, distancing and a lack of personal accomplishment being related to parenting. But even if the structure of the parental burnout measure corresponds with the professional burn out measure it does not necessarily mean that both constructs have similar determinants, consequences or underlying mechanisms. When it comes to parental burnout, the depletion of resources in the parental domains can be caused by too high parental demands in combination with too little personal or contextual resources in the parental domain to prevent a loss of parental resources to occur (cf. Hobfoll, 1989). Future research focusing on theoretically relevant factors linked to parental burnout are certainly needed, like parental personality, intra-family and extra-family support or role salience and career identity versus parental identity (Kossek et al., 2012).

Third, the mean scores of the subscales, however, were lower than the mean scores reported for the subscales and the total PBI score in Roskam et al. (2017). The mean scores in Roskam et al.’s (2017) community sample for each scale were almost twice as high as the mean scores in our Dutch sample. This can be explained by the fact that Roskam et al.’s sample included more ‘special needs children’ with a chronic illness or disability (11.3–13.7%). In our sample only 2.4% of parents reported to have people with a disability or chronic illness in their home to take care of. Previous studies showed that the burnout scores of mothers and fathers of children with a history of chronic or serious diseases were significantly higher compared to those of reference parents (Norberg, 2007; Lindström et al., 2011). The small number of those strained families in our sample may have reduced the level of mean scores.

Fourth, although our sample characteristics were similar to Roskam et al.’s (2017) samples concerning the parents’ age, educational level, and marital status, our sample consisted of 60% women, whereas Roskam’s samples consisted of far higher percentages of women (i.e., 83 and 87%). An interesting finding of the present study representing the Dutch context was that men indicated higher levels of parental burnout, particularly emotional distancing and decreased personal accomplishment regarding parental activities with children and when it comes to the fulfillment of their parental roles in the home domain, whereas women indicated higher levels of work-related burnout and family-to-work-conflict. This may reflect that both men and women may not feel (yet) comfortable with their combined role of working and caring parent. Women’s interruptions by family in the home domain may reflect their care role salience, being concerned with home obligations during work, depleting their time and energy in the work domain. In order to allow their role in the work domain to be dominant, men’s may develop more distance to their home role when home demands increase which is reflected in these two dimensions of parental burnout. This finding can be linked to role theory, which is also used to explain the outcomes of studies, such as those by Cinamon and Rich (2002), Byron (2005), and van Veldhoven and Beijer (2012), which showed that mothers report higher levels of work-family conflict than fathers.

Normative expectations for being a ‘good parent’ still emphasize for women that their primary role is in the family, whereas for men the role of provider is primary, despite the transformations to ‘dual earner-dual carer’ families characteristic of most Western societies today (e.g., Okimoto and Heilman, 2012; Poelmans, 2012; Van Engen et al., 2012). While being a good father is generally not perceived as incompatible with work or a career, research clearly demonstrates ambivalence toward working mothers (e.g., Greenberger et al., 1988; Heilman and Okimoto, 2008; King, 2008). Normative expectations for being a good parent are often internalized, with feelings of ‘work-family guilt’ when parents violate these normative expectations (Morgan and King, 2012). As a result, working mothers tend to report higher feelings of family-to-work conflict whereas men tend to report higher work-to-family conflict as a meta-analysis by Byron (2005) has shown. Also the results of the present study may be explained by the presence of traditional social roles men and women have in society. Although men experience lower levels of work-to-family or family-to-work conflict, they may experience parental burnout because this role is relatively new to them and they might not (yet) have the personal and structural resources in the parental domain to cope with these. Consequently, they may experience feelings of distancing and decreased personal accomplishment when it comes to fulfilling their role as father.

It may also be suggested that at least for Dutch working women, higher levels of family-work conflict make it more likely they will suffer from work-related burnout. This finding is in agreement with gender socialization theory (Barnett and Rivers, 1998) and suggestive of the fact that women must tackle the double burden of work and home (more than men).

The fact that, for men, parental burnout –as reflected in higher levels of emotional distancing and a lack of personal accomplishment- was more prevalent in our study may suggest that men in our sample were less able to cope with the strains of home, that is, they may experience home factors more as home demands (Peeters et al., 2005). Therefore, they may have reported more parental burnout instead of work-related burnout than women.

### Limitations and Recommendations for Future Research

In the presented study, we found evidence for the generalizability of the newly developed scale translated into Dutch to measure parental burnout among Dutch employees as presented by Roskam et al. (2017). Like other studies, however, this study had some limitations. First, a convenience sampling (by students) was used to recruit men and women respondents (combining work and parental activities) for this research. Therefore, the sample might be slightly more homogeneous than if a more random sampling method would have been used (Suri, 2011). Although demographic statistics were similar to the study conducted by Roskam et al. (2017), which allows for some comparison with the French speaking sample, it is necessary to be careful with making generalizations of this sample in general and to the Dutch population in particular. Future research could further validate the tri-dimensional scale for the Dutch context and other national contexts alike. This would be particularly interesting since the prevalence of parental burn-out among men and women working parents may be determined by the national context which may support or may not support working parents, also when it comes to the care for their children.

In a related vein, second, responses on the PBI showed that few people in our sample reported extreme parental burnout and that scores were skewed. It was beyond the scope of this study to verify whether this reflects reality or whether social desirability is an issue involved in measuring parental burnout. A possible effect of the skewed distribution of scores on parental burnout has been minimized by performing transformation analyses to the parental burnout measure. Moreover, in view of the idealism that has emerged the last decennium concerning parenting (i.e., advocating warm, sensitive and supportive parenting, raising children to become empowered people in their own rights), the pressure on parents has increased significantly (Daly, 2007). By a desire to comply with these social standards of being a good parent, people may find it hard to admit that perhaps they do not fully meet this ideal (Morsbach and Prinz, 2006). Therefore, the responses might show a more optimistic picture of the reality, causing an underestimation of the prevalence of parental burnout. In view of this, future research is advised to further address the potential role of social desirability in the assessment of parental burnout.

In conclusion, third, future research could further explore the differences between men and women in parental burnout focusing on the underlying explanatory mechanisms between the phenomenon and its determinants and salient employee outcomes, respectively. In this study, our primary aim was to replicate the study by Roskam et al. (2017). Of course, more factors than distinguished in the current study might affect differences between fathers and mothers in their experiences of parental burnout complaints, such as characteristics of the family context (analogous to the organizational level), parental demands (e.g., the degree of care dependence of the children, associated with age or illness for example) and parental resources (e.g., practical support from partner or others or (the lack of) personality characteristics) (Mikolajczak et al., 2017).

In accordance with Peeters et al. (2005), for example, parental burnout and parenting demands could reflect both the magnitude of quantitative, emotional and mental demands. Future research should disentangle whether there are different antecedents of these demands. For instance, the number of children might be more predictive of perceived quantitative demands as more children means more time and time-related conflicts, whereas the age of children might be more predictive of mental demands. Most parents worry from time to time about their children, yet the health condition of their children can be argued to particularly affect emotional demands on parents. Future research should address in more detail the mechanism that play a role in parental burnout and its antecedents.

### Practical Implications

Now that both fathers and mothers increasingly have to take on a major role in both the work and the parental domains, parental burnout will become an increasingly important topic for individuals, organizations and managers to take into consideration. Just like work-related burnout, parental burnout can lead to absenteeism at work (Maslach and Leiter, 2016).

In addition, it can have detrimental effects on the functioning in households, which may not only have implications for parents and children in the non-work domain, but in the longer run, may also affect the functioning of parents in the work domain. Acknowledgment of the concept of parental burnout and its three dimensions by individuals themselves and other stakeholders in the non-work and work domains may help to recognize the associated problems. More concretely, it can lead to higher levels of informal or informal support from stakeholders inside or outside the parental domain. In addition, it can enable individuals and others to create more opportunities to develop the personal resources that individuals need to balance between the increased parental demands and the needed resources in order to prevent or stop a potential loss-of resources spiral and foster a gain spiral to combat potential negative outcomes associated with parental burnout.

## Ethics Statement

The study was approved by the Tilburg University Ethics Review Board and confidentiality and anonymity was guaranteed. The questionnaire for the respondents enclosed verification and informed consent forms and provided the (potential) respondents with all information about the aims of the study. All participation was voluntary and no participants were minor or vulnerable people.

## Author Contributions

MVE, PP, and HVB conducted the data collection and were responsible for the study design and parts of the manuscript. HVB did the data analysis. All authors contributed to the final version of the paper and provided input for intellectual content.

## Conflict of Interest Statement

The authors declare that the research was conducted in the absence of any commercial or financial relationships that could be construed as a potential conflict of interest.
